# Supplementary material for: HiCImpute: A Bayesian hierarchical model for identifying structural zeros and enhancing single cell Hi-C data
Source: PLoS Comput Biol. 2022 Jun 13;18(6):e1010129. doi: 10.1371/journal.pcbi.1010129 (PMC9232133; doi:10.1371/journal.pcbi.1010129)
Supplement: S1 Text — (PDF) [file pcbi.1010129.s001.pdf]

Supplementary Document for "A Powerful Bayesian Hierarchical Model for Identifying Structural Zeros and Enhancing Single Cell Hi-C Data"

Qing Xie[1], Chenggong Han[1], Victor Jin[2], and Shili Lin[1,3,4,*]

[1]Interdisciplinary Ph.D. Program in Biostatistics, [3]Department of Statistics, [4]Translational Data Analytics Institute, The Ohio State University, Columbus, OH 43210. [2] Department of Molecular Medicine, University of Texas Health Science Center, San Antonio, TX 78229.

Markov chain Monte Carlo Procedure

In the following, we provide the prior specifications, the posterior distributions, the Markov chain Monte Carlo (MCMC) sampling schemes, and convergence diagnostics. The notations are the same as those in the main paper and may not be reintroduced.

To distinguish the SZs from the DOs, we define an indicator variable S_{ij}, which equals to 1 if loci i and j do not interact; otherwise, it is 0. That is, $S_{ij} \sim \text{Bernoulli}(\pi_{ij})$, where π_{ij} is the probability that pair i and j do not interact. This probability, π_{ij}, is assumed to follow a Beta distribution with parameters a_{ij}^{β} and b_{ij}^{β}, and a_{ij}^{β} is further assumed to be uniformly distributed on (1,1000) to account for a large range of possible shapes. The mean of the Beta distribution, $\frac{a_{ij}^{\beta}}{a_{ij}^{\beta}+b_{ij}^{\beta}}$, is governed by the proportion of observed zeros at (i, j), denoted as $\hat{p}_{ij}$. Specifically, we define δ_{ij} to be a uniformly distributed variable that centers at $\hat{p}_{ij}$ with radius ϵ_1 (default is set to be 0.5), and we let $logit(\frac{a_{ij}^{\beta}}{a_{ij}^{\beta}+b_{ij}^{\beta}})$ follow a Normal distribution with mean and standard deviation being $logit(\delta_{ij})$ and σ_δ, respectively. That is, if we observe a

large proportion of zeros for pair i and j, then it is more likely, a priori, that the pair is a structural zero.

We allow for cell-to-cell variability by setting up an additional hierarchy to model μ_{ij}^k as follows: $\mu_{ij}^k \sim \text{Normal}^+(\mu_{ij}, \sigma_{ij}^2)$, where Normal$^+$ is a truncated normal distribution on positive numbers, σ_{ij}^2 is taken to be the standard deviation of nonzero counts in a neighborhood centered at (i, j), and μ_{ij} is further assumed to follow a Gamma distribution with shape and scale parameters being α_{ij} and β_{ij}, respectively. Its mean, $\alpha_{ij}\beta_{ij}$, borrows information from both the bulk Hi-C and the neighborhood data across similar SCs, as already described in the main text. Further, α_{ij} is assumed to follow a Uniform distribution on (1,1000) to allow for a wide variety of shapes for the distribution.

To make inferences about the parameters, we devise a Markov chain Monte Carlo (MCMC) sampling procedure as follows. We first write the posterior distribution of Θ (a vector containing all parameters, including π_{ij}'s and μ_{ij}^k's, the main parameters of interest, as well as nuisance parameters):

$$
\begin{aligned}
P(\Theta|s,y) &\propto P(y|s,\Theta)\times P(s|\Theta)\times P(\Theta)\\
&\propto \prod_{(i,j,k):y_{ijk}>0}\frac{(\lambda^k\mu_{ij}^k)^{y_{ijk}}e^{-\lambda^k\mu_{ij}^k}}{y_{ijk}!}\prod_{(i,j,k):y_{ijk}=0}\left[e^{-\lambda^k\mu_{ij}^k}\right]^{1-s_{ij}}\\
&\times\prod_{i,j}[\pi_{ij}]^{\mathbb{1}\{s_{ij}=1\}}[1-\pi_{ij}]^{\mathbb{1}\{s_{ij}=0\}}\\
&\times\prod_{ij}\prod_{k}\frac{\phi(\frac{\mu_{ij}^k-\mu_{ij}}{\sigma_\mu})}{\sigma_\mu[1-\Phi(-\mu_{ij}/\sigma_\mu)]}\\
&\times\prod_{i,j}(\pi_{ij})^{a_{ij}^t-1}(1-\pi_{ij})^{b_{ij}^t-1}\frac{\Gamma(a_{ij}^t+b_{ij}^t)}{\Gamma(a_{ij}^t)\Gamma(b_{ij}^t)}\\
&\times\prod_{i,j}\frac{1}{1000-1}\mathbb{1}_{\{1\le a_{ij}^t\le 1000\}}\\
&\times\prod_{i,j}exp\{-\frac{1}{2\sigma_\delta^2}(logit(\frac{a_{ij}}{a_{ij}+b_{ij}})-logit(\delta_{ij}))^2\}\frac{1}{\frac{a_{ij}}{a_{ij}+b_{ij}}(1-\frac{a_{ij}}{a_{ij}+b_{ij}})}\\
&\times\prod_{i,j}\frac{1}{min\{\hat{p}+\epsilon_1,1\}-max\{\hat{p}-\epsilon_1,0\}}\mathbb{1}_{\{max\{\hat{p}-\epsilon_1,0\}\le\delta_{ij}\le min\{\hat{p}+\epsilon_1,1\}\}}\\
&\times\prod_{i,j}\frac{1}{\Gamma(\alpha_{ij}^t)(\beta_{ij}^t)^{\alpha_{ij}^t}}\mu_{ij}^{\alpha_{ij}^t-1}e^{-\mu_{ij}/\beta_{ij}^t}\\
&\times\prod_{i,j}\frac{1}{1000-1}\mathbb{1}_{\{1\le\alpha_{ij}^t\le 1000\}}\\
&\times\prod_{i,j}\frac{1}{(B_{ij}+\epsilon_2)-max\{0,B_{ij}-\epsilon_2\}}\mathbb{1}_{\{max\{0,B_{ij}-\epsilon_2\}\le\alpha_{ij}\beta_{ij}\le B_{ij}+\epsilon_2\}}
\end{aligned}
$$

To sample from the posterior distributions of the parameters in Θ, we use Metropolis-Hastings algorithms, and in particular the Gibbs sampler whenever the conditional distribution of a parameter is of a commonly known one. In the following, we briefly describe the

updating schemes. We first note that Θ_{-g} denote the subvector of Θ that includes all the parameters except g.

- Update α_{ij}^t:

  Using the current α_{ij}^t, sample a candidate α_{ij}^{t*} from the proposal distribution $J_{\alpha_{ij}}(\alpha_{ij}^{t*}|\alpha_{ij}^t)$, a Uniform$(1, 1000)$ distribution, and calculate the ratio of the densities,

$$r = \frac{p(\alpha_{ij}^{t*}|y, \Theta_{-\alpha_{ij}^t})}{p(\alpha_{ij}^t|y, \Theta_{-\alpha_{ij}^t})}$$

  where

$$p(\alpha_{ij}^{t*}|y, \Theta_{-\alpha_{ij}^t}) \propto \frac{1}{\Gamma(\alpha_{ij}^t)(\beta_{ij}^t)^{\alpha_{ij}^t}} \mu_{ij}^{\alpha_{ij}^t - 1} e^{-\mu_{ij}/\beta_{ij}} \mathbb{1}_{\{0 \le \alpha_{ij} \le 1000\}} \mathbb{1}_{\{max\{0, B_{ij} - \epsilon_2\} \le \alpha_{ij}\beta_{ij} \le B_{ij} + \epsilon_2\}}$$

  Accept α_{ij}^{t*} with probability $min(r, 1)$.

- Update β_{ij}:

  Sample μ_{ij} from a Uniform$(\max\{0, B_{ij} - \epsilon_2\}, B_{ij} + \epsilon_2)$ distribution, and solve for β_{ij} using $\alpha_{ij}\beta_{ij} = \mu_{ij}$.

- Update μ_{ij}:

  Using the current μ_{ij}^t, sample a candidate μ_{ij}^{t*} from the proposal distribution $J_{\mu_{ij}}(\mu_{ij}^{t*}|\mu_{ij}^t)$, a $Normal^+(\mu_{ij}^t, 0.5)$ distribution, and calculate the ratio of the densities,

$$r = \frac{p(\mu_{ij}^{t*}|y, \Theta_{-\mu_{ij}^t})}{p(\mu_{ij}^t|y, \Theta_{-\mu_{ij}^t})}$$

where

$$p(\mu_{ij}|y,\Theta_{-\mu_{ij}^t}) \propto \mu_{ij}^{\alpha_{ij}-1}e^{-\mu_{ij}/\beta_{ij}}\prod_{k=1}^{100}\frac{\phi(\mu_{ij}^k-\mu_{ij})/\sigma_\mu)}{\sigma_\mu[1-\Phi(-\mu_{ij}/\sigma_\mu)]},$$

and ϕ,Φ are the pdf and cdf of the standard normal distribution, respectively. Accept μ_{ij}^{t*} with probability $min(r,1)$.

- Update $\mu_{ij}^k(k=1,2,\cdots,100)$:

Using the current μ_{ij}^k, sample a candidate μ_{ij}^{k*} from the proposal distribution $J_{\mu_{ij}^k}(\mu_{ij}^{k*}|\mu_{ij}^k)$, a $Normal^+(\mu_{ij}^k,0.5)$ distribution, and calculate the ratio of the densities,

$$r=\frac{p(\mu_{ij}^{k*}|y,\Theta_{-\mu_{ij}^k})}{p(\mu_{ij}^k|y,\Theta_{-\mu_{ij}^k})},$$

where

$$p(\mu_{ij}|y,\Theta_{-\mu_{ij}^k}) \propto \left[(\mu_{ij}^k)^{y_{ijk}}e^{-\lambda^k\mu_{ij}^k}\right]^{\mathbb{1}_{y_{ijk}>0}} \times \left[e^{-\lambda^k\mu_{ij}^k}\right]^{\mathbb{1}_{s_{ij}=0}\mathbb{1}_{y_{ijk}=0}}\phi(\frac{\mu_{ij}^k-\mu_{ij}}{\sigma_\mu})$$

and ϕ is the pdf of the standard normal distribution. Accept μ_{ij}^{k*} with probability $min(r,1)$.

- Update a_{ij}

Using the current a_{ij}, sample a candidate a_{ij}^{t*} from the proposal distribution $J_{a_{ij}}(a_{ij}^{t*}|a_{ij})$, Uniform$(1,1000)$, and calculate the ratio of the densities,

$$r=\frac{p(a_{ij}^{t*}|y,\Theta_{-a_{ij}})}{p(a_{ij}|y,\Theta_{-a_{ij}})}$$

where

$$p(a_{ij}^{t*}|y,\Theta_{-a_{ij}}) \propto \pi_{ij}^{a_{ij}-1}(1-\pi_{ij})^{b_{ij}-1}\frac{\Gamma(a_{ij}+b_{ij})}{\Gamma(a_{ij})}\mathbb{1}_{\{0\ \leq a_{ij}\leq A_1\}}$$

and

$$exp\{\frac{1}{2\sigma_\delta^2}(logit(\frac{a_{ij}}{a_{ij}+b_{ij}})-logit(\delta_{ij}))^2\}\frac{1}{\frac{a_{ij}^\beta}{a_{ij}+b_{ij}}(1-\frac{a_{ij}}{a_{ij}^\beta+b_{ij}})}.$$

Accept a_{ij}^{t*} with probability $min(r,1)$.

- Update δ_{ij}:

Using the current δ_{ij}, sample a candidate δ_{ij}^{t*} from the proposal distribution $J_{\delta_{ij}}(\delta_{ij}^{t*}|\delta_{ij})$, a uniform($max\{0,\hat{p}_{ij}-\epsilon_1\},min\{\hat{p}_{ij}+\epsilon_1\}$) distribution, and calculate the ratio of the densities,

$$r=\frac{p(\delta_{ij}^{t*}|y,\Theta_{-\delta_{ij}})}{p(\delta_{ij}|y,\Theta_{-\delta_{ij}})}$$

where

$$p(logit(\delta_{ij})|B,S) \propto exp\{-\frac{1}{2\sigma_\delta^2}(logit(\frac{a_{ij}}{a_{ij}+b_{ij}})-logit(\delta_{ij}))^2\}\mathbb{1}_{\{max\{\hat{p}-\epsilon_1,0\}\leq\delta_{ij}\leq min\{\hat{p}+\epsilon_1,1\}\}}.$$

Accept δ_{ij}^{t*} with probability $min(r,1)$.

- Update b_{ij}:

Solve for b_{ij}, using $logit(\frac{a_{ij}}{a_{ij}+b_{ij}})=logit(\delta_{ij})$.

- Update π_{ij}

  Sample π_{ij}^{t+1} from $Beta(\mathbb{1}_{\{s_{ij}=1\}} + a_{ij}, \mathbb{1}_{\{s_{ij}=0\}} + b_{ij})$ because

$$p(\pi_{ij}|B, S) \propto \pi_{ij}^{\mathbb{1}_{\{s_{ij}=1\}}+a_{ij}-1}(1-\pi_{ij})^{\mathbb{1}_{\{s_{ij}=0\}}+b_{ij}-1}.$$

- Update s_{ij}

  Sample s_{ij}^{t+1} from $Bernoulli(\frac{\pi_{ij}}{\pi_{ij}+(1-\pi_{ij})e^{-\sum_{k:y_{ijk}=0}\lambda^k\mu_{ij}^k}})$ because

$$p(s_{ij}|B, S) \propto [\pi_{ij}]^{s_{ij}}[1-\pi_{ij}]^{1-s_{ij}}[e^{-\sum_{k:y_{ijk}=0}\lambda^k\mu_{ij}^k}]^{1-s_{ij}}.$$

Convergence diagnostics. Trace plot, density plot, and cumulative mean plot were drawn to assess the performance of MCMC. An example cumulative mean plots for several parameters are provided to show that the chains converged property with stable estimates of the parameter values (Supplementary Figure S8). We also consider the Gelman–Rubin diagnostic by analyzing the difference between multiple Markov chains [2, 1]. Starting from different points, the three chains converged ultimately, and the scale reduction factors are all less than 1.1 for all the settings considered, indicating convergence. As an example, we show the trace plots and density plots for several parameters for the dataset with 10 single cells from T1 at 7K sequencing depth, which shows that the three chains are well mixed, consistent with the conclusion from considering the reduction factors (Figure S9). As a further evidence that our MCMC algorithm works well, we also provide an example to show that the autocorrelations for multiple parameters decay at a reasonable rate, as one would expect for a well-mixing chain (Figure S10).

References

[1] S. P. Brooks and A. Gelman. General methods for monitoring convergence of iterative simulations. *Journal of computational and graphical statistics*, 7(4):434–455, 1998.

[2] A. Gelman, D. B. Rubin, et al. Inference from iterative simulation using multiple sequences. *Statistical science*, 7(4):457–472, 1992.

[3] H. Hong, S. Jiang, H. Li, G. Du, Y. Sun, H. Tao, C. Quan, C. Zhao, R. Li, W. Li, et al. Deephic: A generative adversarial network for enhancing hi-c data resolution. *PLoS computational biology*, 16(2):e1007287, 2020.

[4] O. Ursu, N. Boley, M. Taranova, Y. R. Wang, G. G. Yardimci, W. Stafford Noble, and A. Kundaje. Genomedisco: A concordance score for chromosome conformation capture experiments using random walks on contact map graphs. *Bioinformatics*, 34(16):2701–2707, 2018.

[5] T. Yang, F. Zhang, G. G. Yardımcı, F. Song, R. C. Hardison, W. S. Noble, F. Yue, and Q. Li. Hicrep: assessing the reproducibility of hi-c data using a stratum-adjusted correlation coefficient. *Genome research*, 27(11):1939–1949, 2017.

[6] M. Yu, A. Abnousi, Y. Zhang, G. Li, L. Lee, Z. Chen, R. Fang, J. Wen, Q. Sun, Y. Li, et al. Snaphic: a computational pipeline to map chromatin contacts from single cell hi-c data. *bioRxiv*, 2020.

[7] Y. Zhang, L. An, J. Xu, B. Zhang, W. J. Zheng, M. Hu, J. Tang, and F. Yue. En-

hancing hi-c data resolution with deep convolutional neural network hicplus. *Nature communications*, 9(1):750, 2018.

[8] J. Zhou, J. Ma, Y. Chen, C. Cheng, B. Bao, J. Peng, T. J. Sejnowski, J. R. Dixon, and J. R. Ecker. Robust single-cell hi-c clustering by convolution-and random-walk–based imputation. *Proceedings of the National Academy of Sciences*, page 201901423, 2019.

[9] H. Zhu and Z. Wang. Scl: a lattice-based approach to infer 3d chromosome structures from single-cell hi-c data. *Bioinformatics*, 35(20):3981–3988, 2019.
