# Supplementary material for: HiCImpute: A Bayesian hierarchical model for identifying structural zeros and enhancing single cell Hi-C data
Source: PLoS Comput Biol. 2022 Jun 13;18(6):e1010129. doi: 10.1371/journal.pcbi.1010129 (PMC9232133; doi:10.1371/journal.pcbi.1010129)
Supplement: S1 Fig — (PDF) [file pcbi.1010129.s002.pdf]

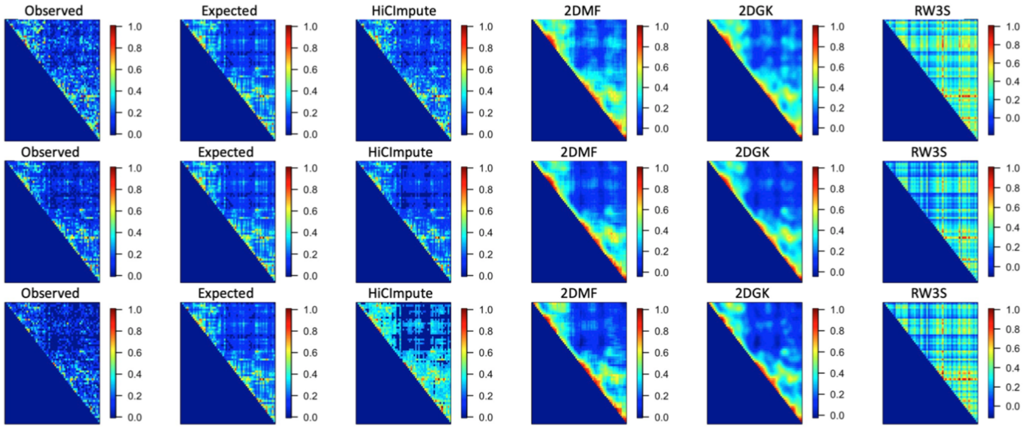

(a) T1, 7k (top), 4k (middle), and 2k (bottom)

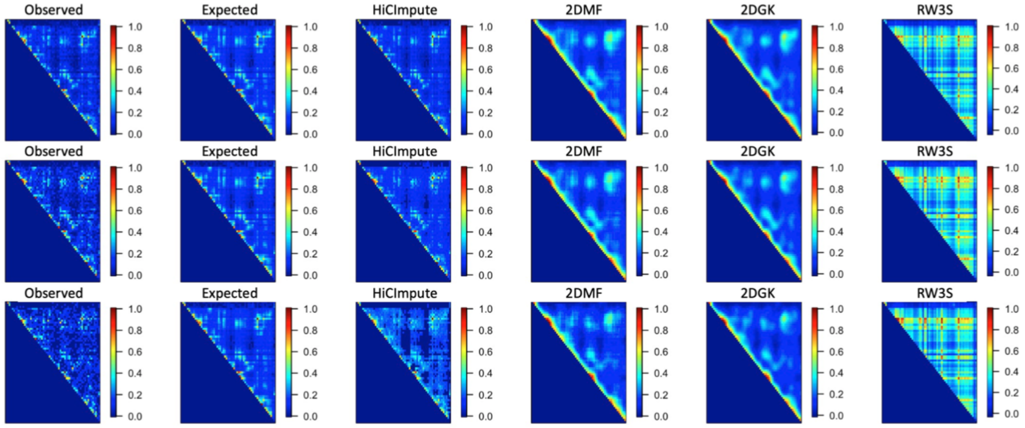

(b) T2, 7k (top), 4k (middle), and 2k (bottom)

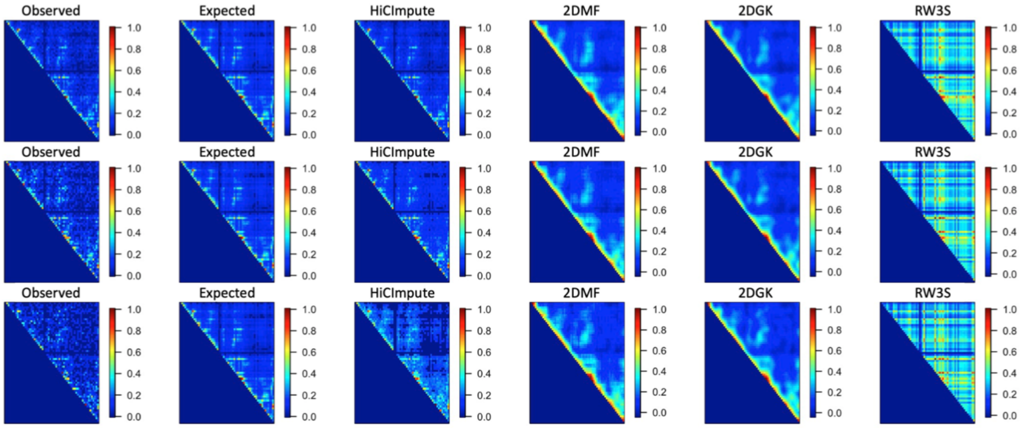

(c) T3, 7k (top), 4k (middle), and 2k (bottom)

Figure S1: Heatmap showing the observed and true (expected) 2D matrix images as well as the results from HiCImpute, 2DMF, 2DGK, and RW3S for T1 (a), T2 (b), and T3 (c) cells at 7K (top), 4K (middle) and 2K (bottom) sequencing depth.
