# Supplementary material for: HiCImpute: A Bayesian hierarchical model for identifying structural zeros and enhancing single cell Hi-C data
Source: PLoS Comput Biol. 2022 Jun 13;18(6):e1010129. doi: 10.1371/journal.pcbi.1010129 (PMC9232133; doi:10.1371/journal.pcbi.1010129)
Supplement: S2 Fig — (PDF) [file pcbi.1010129.s003.pdf]

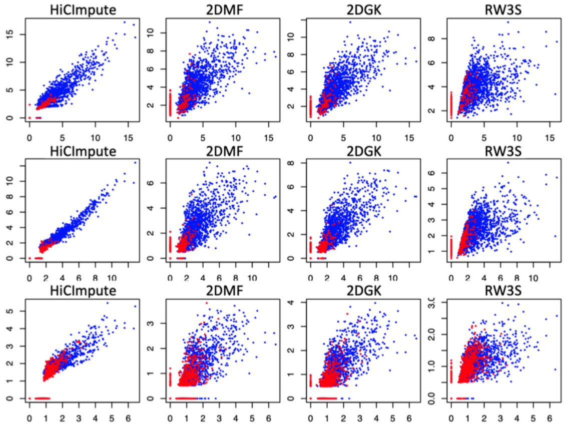

(a) T1, 7k (top), 4k (middle), and 2k (bottom)

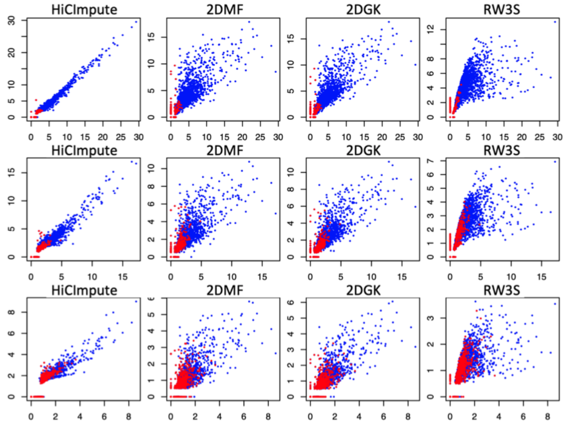

(b) T2, 7k (top), 4k (middle), and 2k (bottom)

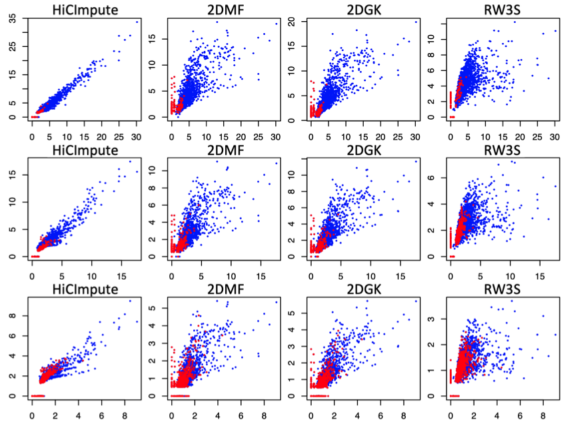

(c) T3, 7k (top), 4k (middle), and 2k (bottom)

Figure S2: Scatterplots of Extected Versus Imputed (SEVI plots) for HiCImpute, 2DMF, 2DGK, and RW3S for T1 (a), T2 (b), and T3 (c) cells at 7K (top), 4K (middle) and 2K (bottom) sequencing depth – the red dots represent the observed zeros, which contain both true SZs and DOs.
