# Supplementary material for: HiCImpute: A Bayesian hierarchical model for identifying structural zeros and enhancing single cell Hi-C data
Source: PLoS Comput Biol. 2022 Jun 13;18(6):e1010129. doi: 10.1371/journal.pcbi.1010129 (PMC9232133; doi:10.1371/journal.pcbi.1010129)
Supplement: S3 Fig — (PDF) [file pcbi.1010129.s004.pdf]

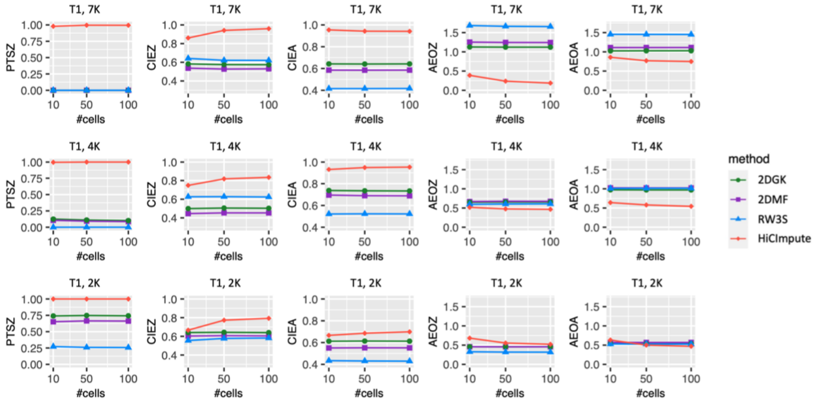

(a) T1, 7k (top), 4k (middle), and 2k (bottom)

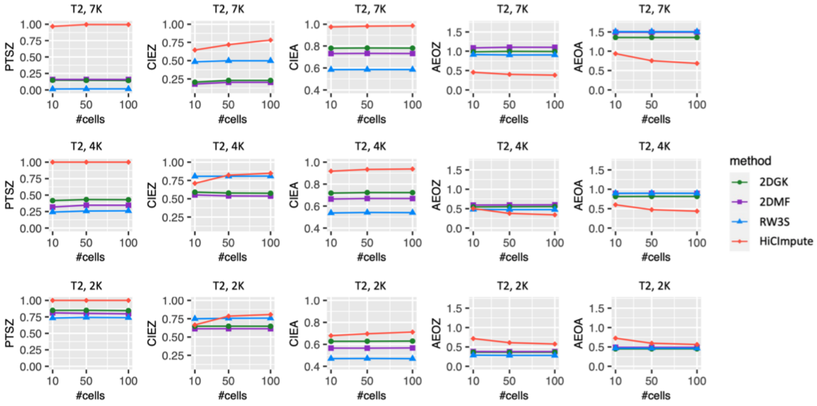

(b) T2, 7k (top), 4k (middle), and 2k (bottom)

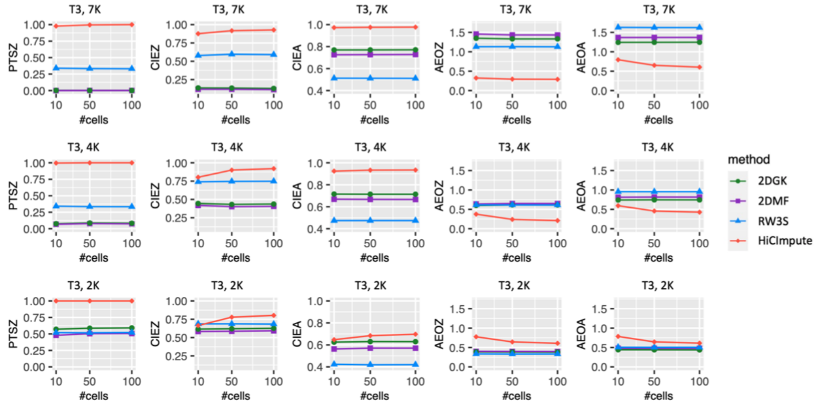

(c) T3, 7k (top), 4k (middle), and 2k (bottom)

Figure S3: Aggregate results (over single cells) based on several evaluation criteria for T1 (a), T2 (b), and T3 (c) cells at 7K (top), 4K (middle) and 2K (bottom) sequencing depth.
