# Supplementary material for: HiCImpute: A Bayesian hierarchical model for identifying structural zeros and enhancing single cell Hi-C data
Source: PLoS Comput Biol. 2022 Jun 13;18(6):e1010129. doi: 10.1371/journal.pcbi.1010129 (PMC9232133; doi:10.1371/journal.pcbi.1010129)
Supplement: S4 Fig — (PDF) [file pcbi.1010129.s005.pdf]

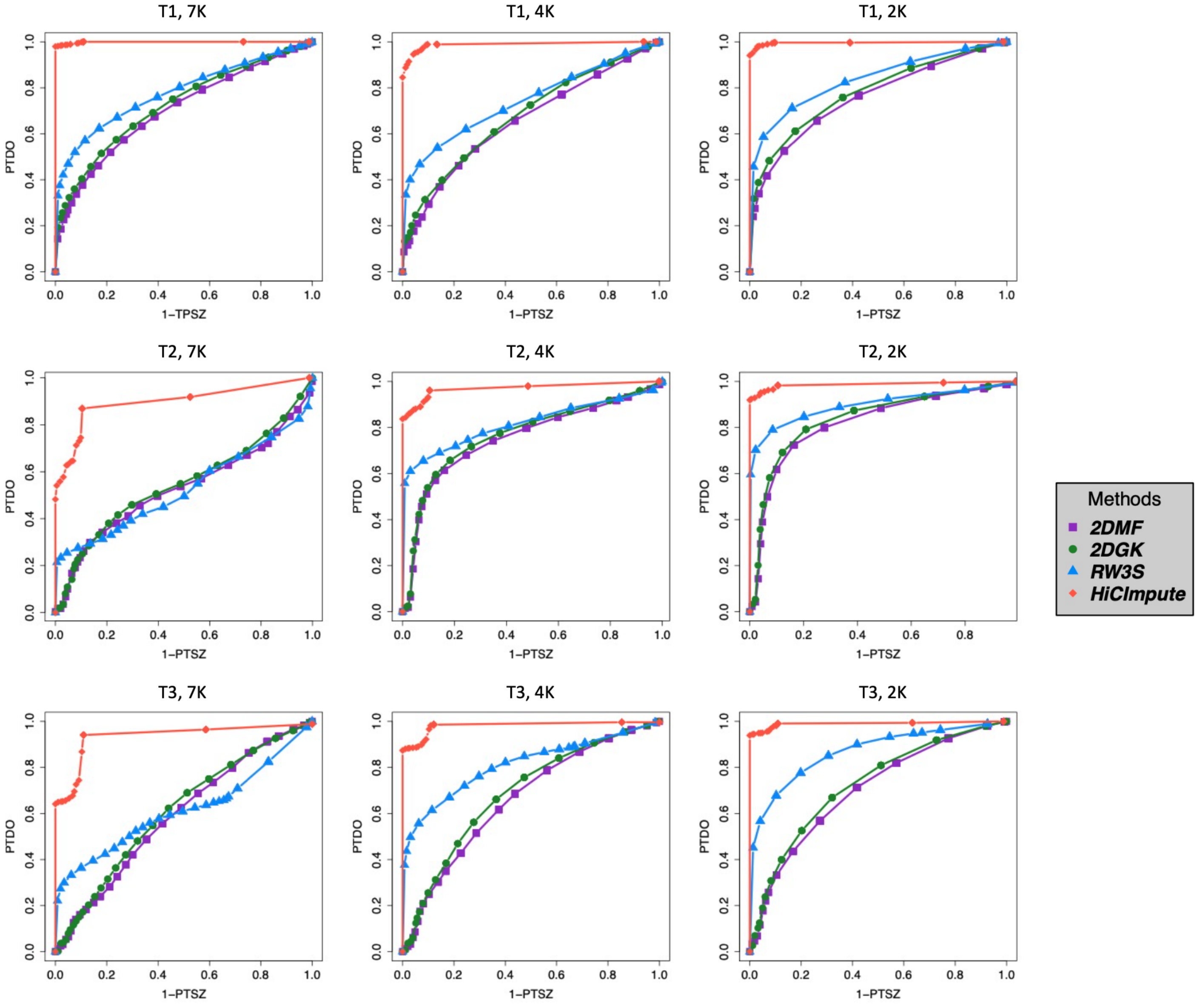

Figure S4: ROC curves accounting for both specificity and sensitivity of HiCImpute, 2DMF, 2DGK, and RW3S for T1 (row1), T2 (row2), and T3 (row 3) cells at 7K (column1), 4K (column2) and 2K (column 3) sequencing depth.
