# Supplementary material for: HiCImpute: A Bayesian hierarchical model for identifying structural zeros and enhancing single cell Hi-C data
Source: PLoS Comput Biol. 2022 Jun 13;18(6):e1010129. doi: 10.1371/journal.pcbi.1010129 (PMC9232133; doi:10.1371/journal.pcbi.1010129)
Supplement: S5 Fig — (a) GM (top row) and PMBC (bottom row); (b) K562A (top) and K562B (bottom); (c) L4 (top) and L5 (bottom). (PDF) [file pcbi.1010129.s006.pdf]

(a)

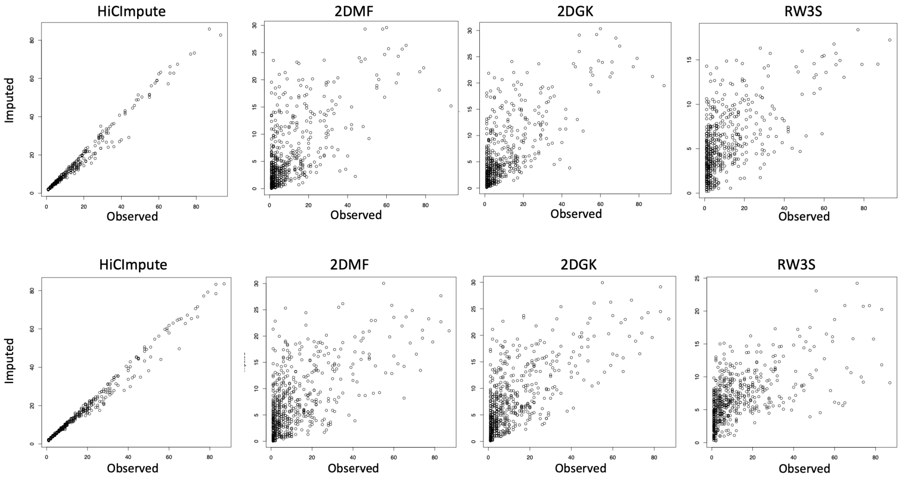

(b)

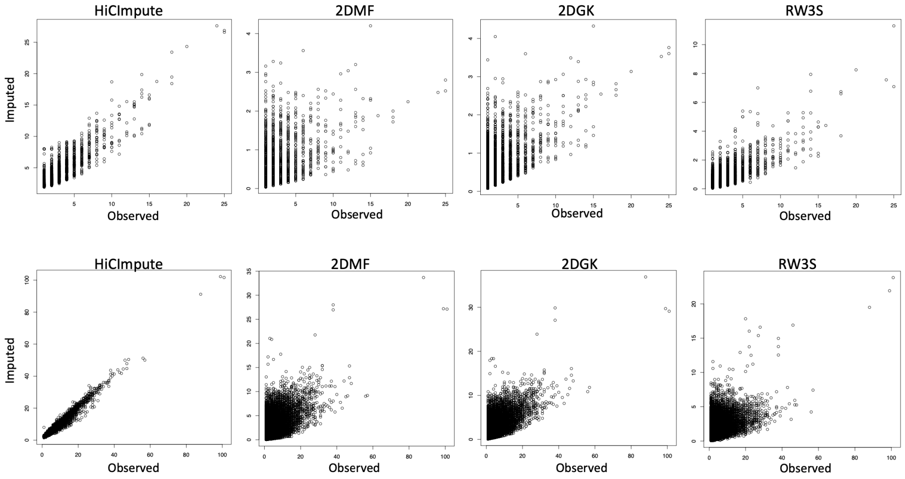

(c)

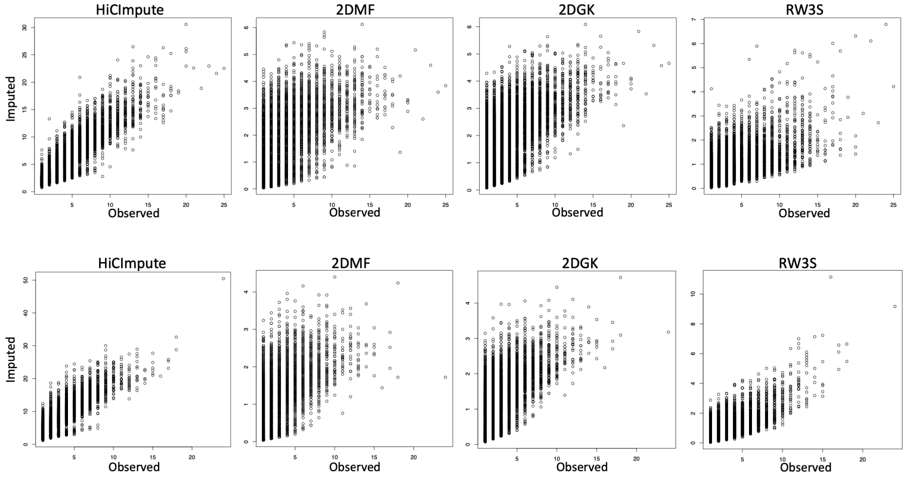

Figure S5: Scatterplots of observed versus imputed (SOVI plots) from HiCImpute, 2DMF, 2DGK, and RW3S. (a) GM (top row) and PMBC (bottom row); (b) K562A (top) and K562B (bottom); (c) L4 (top) and L5 (bottom).
