# Supplementary material for: HiCImpute: A Bayesian hierarchical model for identifying structural zeros and enhancing single cell Hi-C data
Source: PLoS Comput Biol. 2022 Jun 13;18(6):e1010129. doi: 10.1371/journal.pcbi.1010129 (PMC9232133; doi:10.1371/journal.pcbi.1010129)
Supplement: S6 Fig — (PDF) [file pcbi.1010129.s007.pdf]

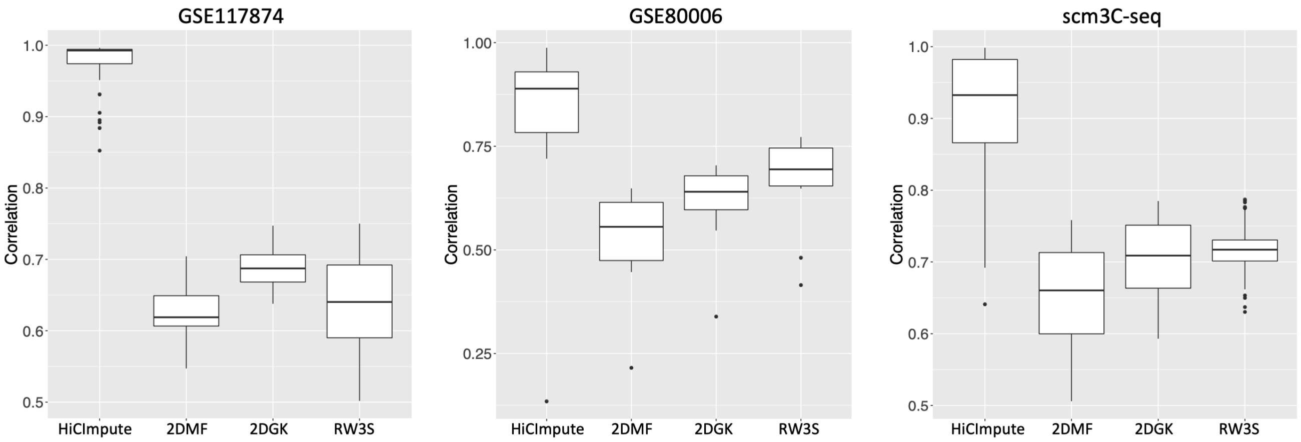

Figure S6: Boxplot of correlations between the observed and imputed from four methods for three datasets: GSE117874 (left), GSE80006 (middle), and scm3C-seq (right).
