# Supplementary material for: HiCImpute: A Bayesian hierarchical model for identifying structural zeros and enhancing single cell Hi-C data
Source: PLoS Comput Biol. 2022 Jun 13;18(6):e1010129. doi: 10.1371/journal.pcbi.1010129 (PMC9232133; doi:10.1371/journal.pcbi.1010129)
Supplement: S7 Fig — The dendrogram was generated using the “complete” method. (PDF) [file pcbi.1010129.s008.pdf]

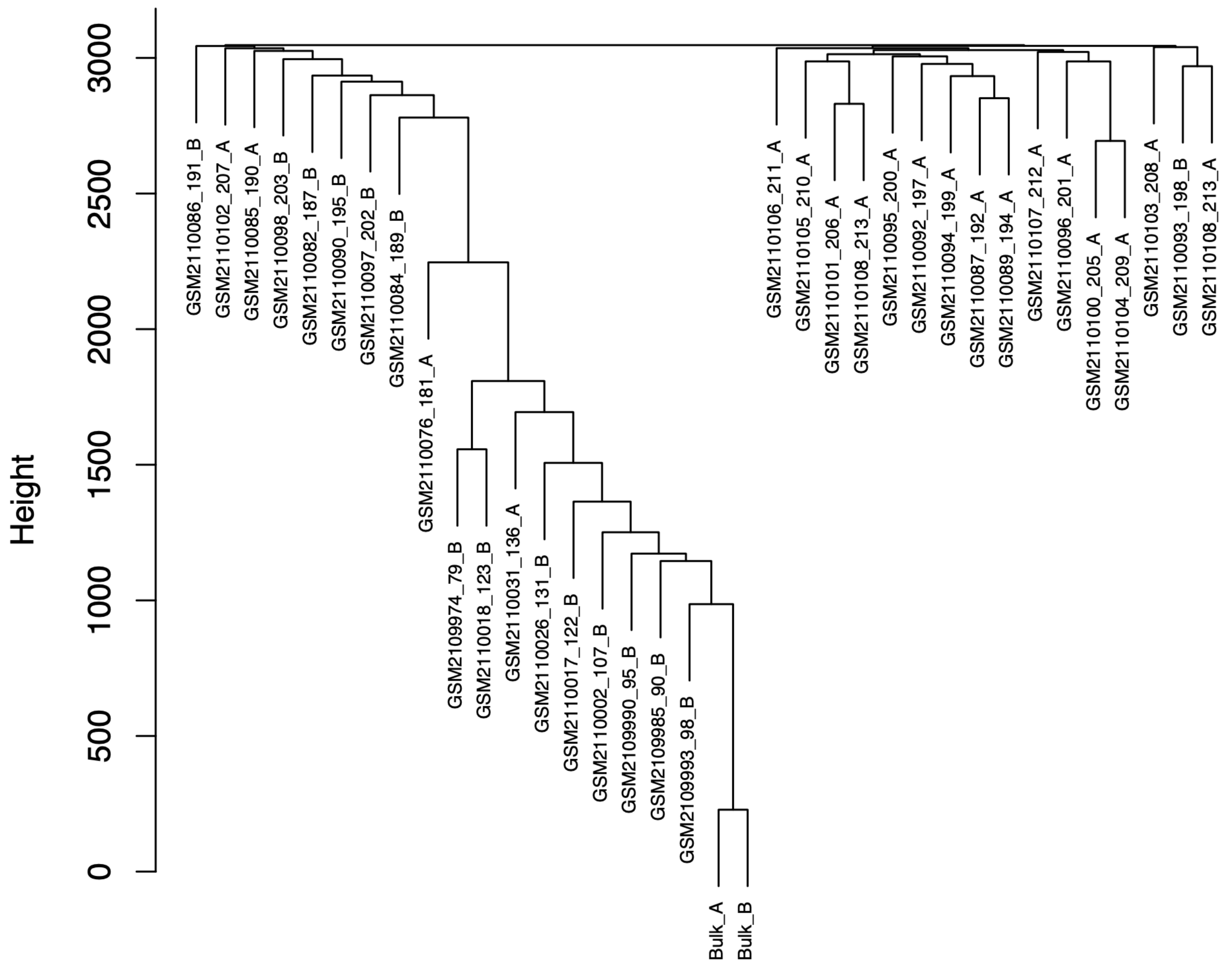

Figure S7: Dendrograms of 34 observed K562 single cells Hi-C data and two bulk datasets. The dendrogram was generated using the "complete" method.
