# Supplementary material for: HiCImpute: A Bayesian hierarchical model for identifying structural zeros and enhancing single cell Hi-C data
Source: PLoS Comput Biol. 2022 Jun 13;18(6):e1010129. doi: 10.1371/journal.pcbi.1010129 (PMC9232133; doi:10.1371/journal.pcbi.1010129)
Supplement: S8 Fig — Recall that a is the shape parameter of the Beta distribution that is the prior of πij; α is the shape parameter of Gamma distribution, which is the prior of μij; μ is the mean of μijk; and π is the probability that the pair is a structural zero. (PDF) [file pcbi.1010129.s009.pdf]

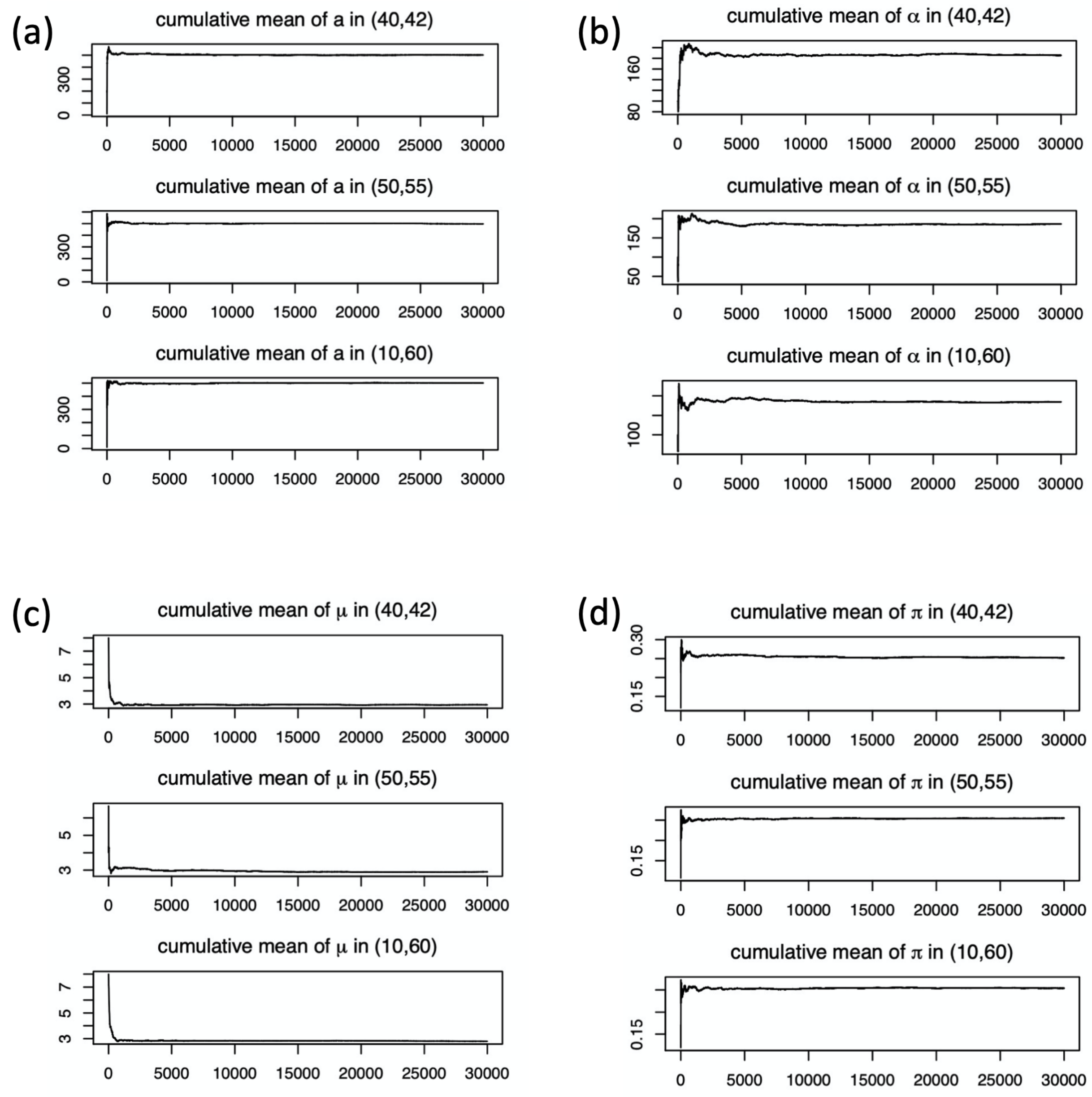

Figure S8: Cumulative mean plots of parameters a (a), α (b), μ (c) and π (d) at 3 positions of a dataset with 10 T1 cells at sequence depth 7k. Recall that a is the shape parameter of the Beta distribution that is the prior of π_{ij}; α is the shape parameter of Gamma distribution, which is the prior of μ_{ij}; μ is the mean of μ_{ij}^{k}; and π is the probability that the pair is a structural zero.
