# Supplementary material for: HiCImpute: A Bayesian hierarchical model for identifying structural zeros and enhancing single cell Hi-C data
Source: PLoS Comput Biol. 2022 Jun 13;18(6):e1010129. doi: 10.1371/journal.pcbi.1010129 (PMC9232133; doi:10.1371/journal.pcbi.1010129)
Supplement: S9 Fig — (PDF) [file pcbi.1010129.s010.pdf]

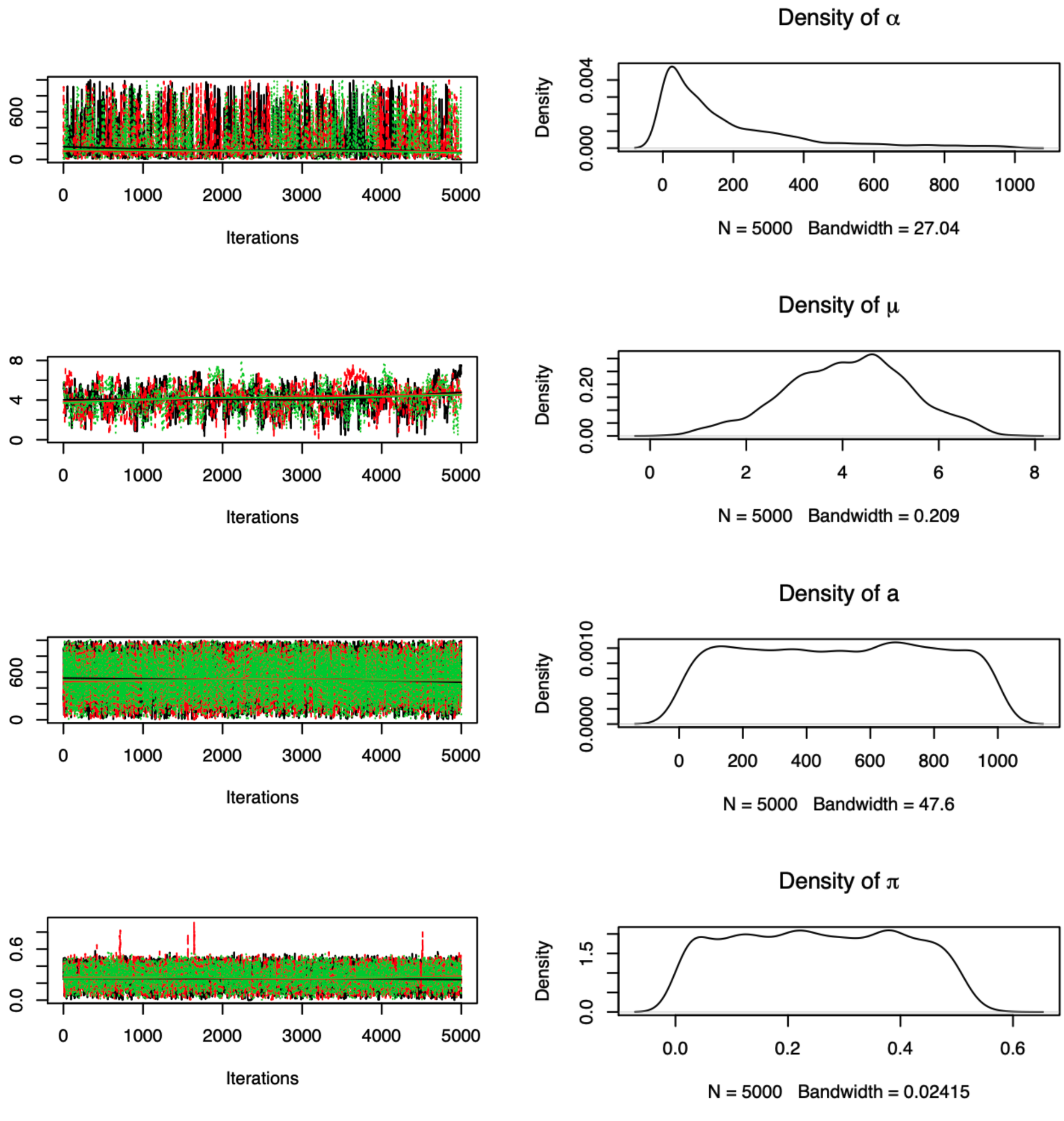

Figure S9: Trace plots of three chains starting from different points and the density of the parameters in the first chain for several parameters.
