# Supplementary material for: HiCImpute: A Bayesian hierarchical model for identifying structural zeros and enhancing single cell Hi-C data
Source: PLoS Comput Biol. 2022 Jun 13;18(6):e1010129. doi: 10.1371/journal.pcbi.1010129 (PMC9232133; doi:10.1371/journal.pcbi.1010129)
Supplement: S10 Fig — (PDF) [file pcbi.1010129.s011.pdf]

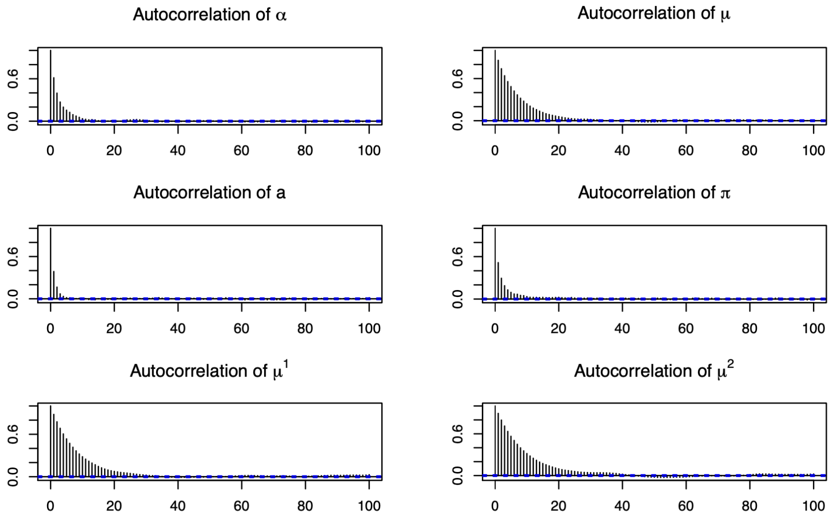

Figure S10: Autocorrelation plots for 6 parameters at position $(i, j) = (40, 42)$ for the simulated dataset with 10 T1 cells at 7K sequencing depth: μ is the overall expectation for all single cells, and μ^1 and μ^2 are the realizations in the first and second single cell, respectively.
