# Supplementary figures and images for: HiCImpute: A Bayesian hierarchical model for identifying structural zeros and enhancing single cell Hi-C data
Source: PLoS Comput Biol. 2022 Jun 13;18(6):e1010129. doi: 10.1371/journal.pcbi.1010129 (PMC9232133; doi:10.1371/journal.pcbi.1010129)

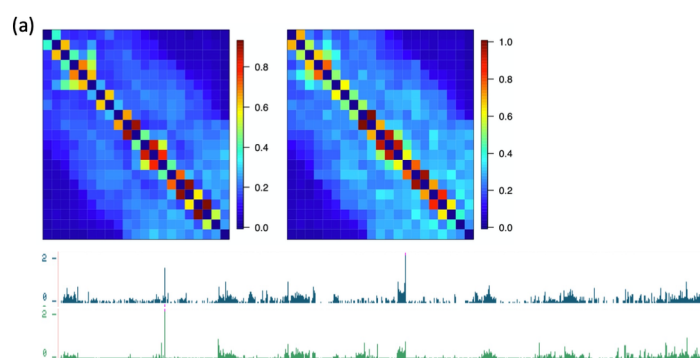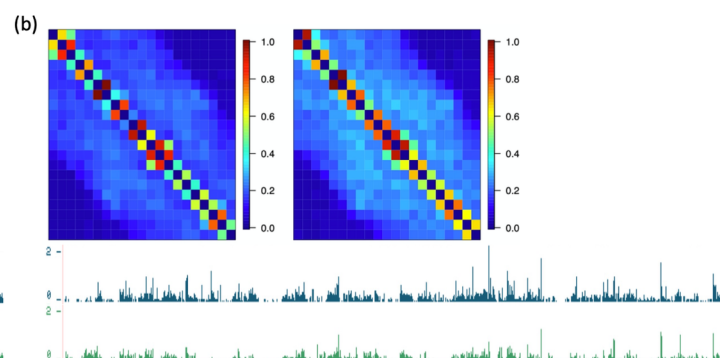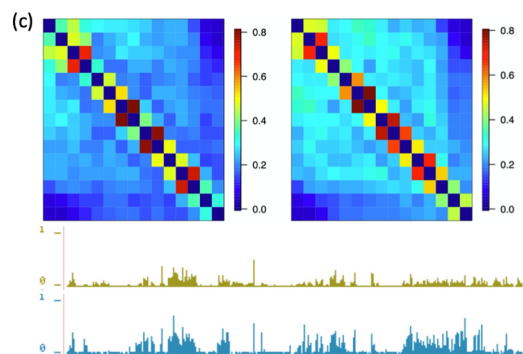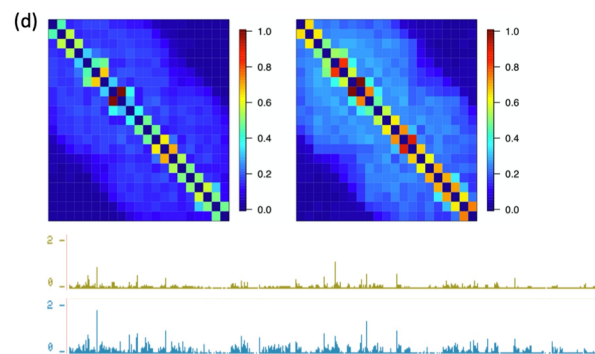

Supplement: S11 Fig — (a) L4 subtype1 (left) and subtype2 (right) on chr8:127,000,000-147,000,000, along with the mean RNA-seq on the same region; (b) L4 subtype1 (left) and subtype2 (right) on chr11:105,000,000-125,000,000, along with the mean RNA-seq on the same region; (c) L5 subtype1 (left) and subtype2 (right) on chr18:1,000,000-15,000,000, along with the mean RNA-seq on the same region; (d) L5 subtype1 (left) and subtype2 (right) on chr20:35,000,000-55,000,000, along with the mean RNA-seq on the same region. (PDF) [file pcbi.1010129.s012.pdf]
