# Supplementary material for: HiCImpute: A Bayesian hierarchical model for identifying structural zeros and enhancing single cell Hi-C data
Source: PLoS Comput Biol. 2022 Jun 13;18(6):e1010129. doi: 10.1371/journal.pcbi.1010129 (PMC9232133; doi:10.1371/journal.pcbi.1010129)
Supplement: S1 Table — (PDF) [file pcbi.1010129.s013.pdf]

Supplementary Tables and Figures

Table S1: Partial list of existing methods for Hi-C data quality improvement.

Method	Goal	Hi-C Type	Category	Ref.
HiCRep	Reproducibility	bulk	Kernel smootthing	[5]
SCL	3D Structure	single cells	Kernel smoothing	[9]
scHiCluster	Clustering	single cells	Kernel smoothing	[8]
scHiCluster	Clustering	single cells	Random walk	[8]
GenomeDISCO	Reproducibility	bulk	Random walk	[4]
SnapHi-C	Chromatin contacts	single cells	Random Walk	[6]
HiCPlus	Data Resolution	bulk	Neural network	[7]
DeepHiC	Data Resolution	bulk	Neural network	[3]
