# Supplementary material for: HiCImpute: A Bayesian hierarchical model for identifying structural zeros and enhancing single cell Hi-C data
Source: PLoS Comput Biol. 2022 Jun 13;18(6):e1010129. doi: 10.1371/journal.pcbi.1010129 (PMC9232133; doi:10.1371/journal.pcbi.1010129)
Supplement: S2 Table — (PDF) [file pcbi.1010129.s014.pdf]

Table S2: Mean (standard error) of the proportion of true dropouts (PTDO) correctly detected by HiCImpute when the detection rate for the proportion of true structural zeros (PTSZ) is set to be 0.95 – stability of results with different neighborhoods*.

Type	Sequence depth	#cells	Neighborhood 1	Neighborhood 2	Neighborhood 3
T1	7k	10	0.98 (0.01)	0.98(0.01)	0.98(0.01)
		50	0.99 (0.01)	0.99(0.01)	0.99(0.01)
		100	0.99 (0.01)	0.99(0.01)	0.99(0.01)
	4k	10	0.95 (0.01)	0.94(0.01)	0.93(0.01)
		50	0.95 (0.01)	0.95(0.01)	0.95(0.01)
		100	0.95 (0.01)	0.95(0.01)	0.95(0.01)
	2k	10	0.95 (0.00)	0.92(0.00)	0.94(0.00)
		50	0.98 (0.00)	0.98(0.00)	0.98(0.00)
		100	0.98 (0.00)	0.99(0.00)	0.99(0.00)
T2	7k	10	0.60 (0.03)	0.59(0.03)	0.58(0.03)
		50	0.64 (0.04)	0.63(0.04)	0.64(0.04)
		100	0.63 (0.04)	0.63(0.04)	0.64(0.04)
	4k	10	0.89 (0.01)	0.89(0.01)	0.90(0.01)
		50	0.88 (0.01)	0.88(0.01)	0.88(0.01)
		100	0.88 (0.01)	0.88(0.01)	0.88(0.01)
	2k	10	0.93 (0.00)	0.93(0.00)	0.92(0.00)
		50	0.95 (0.00)	0.95(0.00)	0.95(0.00)
		100	0.96 (0.00)	0.95(0.00)	0.95(0.00)
T3	7k	10	0.67 (0.02)	0.68(0.02)	0.67(0.02)
		50	0.66 (0.03)	0.66(0.03)	0.66(0.03)
		100	0.67 (0.03)	0.67(0.03)	0.67(0.03)
	4k	10	0.91 (0.01)	0.91(0.01)	0.91(0.01)
		50	0.89 (0.01)	0.89(0.01)	0.89(0.01)
		100	0.89 (0.01)	0.89(0.01)	0.89(0.01)
	2k	10	0.96 (0.00)	0.96(0.00)	0.96(0.00)
		50	0.96 (0.00)	0.95(0.00)	0.96(0.00)
		100	0.95 (0.00)	0.95(0.00)	0.95(0.00)

*Neighborhood 1: up to 24 closet neighbors, as indicated in Figure 1 of the main text; Neighborhood 2: up to 8 closet neighbors; Neighborhood 3: up to 12 first- and second-degree neighbors as typically defined in a square lattice.
