# Supplementary material for: HiCImpute: A Bayesian hierarchical model for identifying structural zeros and enhancing single cell Hi-C data
Source: PLoS Comput Biol. 2022 Jun 13;18(6):e1010129. doi: 10.1371/journal.pcbi.1010129 (PMC9232133; doi:10.1371/journal.pcbi.1010129)
Supplement: S3 Table — (PDF) [file pcbi.1010129.s015.pdf]

Table S3: Mean (standard error) of the proportion of true dropouts (PTDO) correctly detected when the detection rate for the proportion of true structural zeros (PTSZ) is set to be 0.95 – robustness to underlying cell-type structures.

Type	Sequence depth	#cells	HiCImpute	2DMF	2DGK	RW3S
T1	7k	10	0.98 (0.01)	0.29 (0.04)	0.31 (0.04)	0.50 (0.06)
		50	0.99 (0.01)	0.27 (0.05)	0.31 (0.05)	0.47 (0.07)
		100	0.99 (0.01)	0.27 (0.04)	0.30 (0.05)	0.46 (0.06)
	4k	10	0.95 (0.01)	0.21 (0.03)	0.24 (0.03)	0.43 (0.03)
		50	0.95 (0.01)	0.18 (0.03)	0.25 (0.03)	0.44 (0.03)
		100	0.95 (0.01)	0.19 (0.03)	0.26 (0.03)	0.44 (0.03)
	2k	10	0.98 (0.00)	0.39 (0.02)	0.45 (0.02)	0.55 (0.02)
		50	0.99 (0.00)	0.39 (0.02)	0.45 (0.02)	0.56 (0.03)
		100	0.98 (0.0)	0.39 (0.02)	0.44 (0.02)	0.56 (0.03)
T2	7k	10	0.61 (0.03)	0.08 (0.03)	0.10 (0.04)	0.26 (0.06)
		50	0.63 (0.04)	0.10 (0.04)	0.11 (0.04)	0.25 (0.05)
		100	0.63 (0.04)	0.10 (0.03)	0.11 (0.03)	0.26 (0.05)
	4k	10	0.89 (0.01)	0.30 (0.02)	0.34 (0.02)	0.63 (0.03)
		50	0.88 (0.01)	0.29 (0.02)	0.33 (0.02)	0.62 (0.03)
		100	0.88 (0.01)	0.29 (0.02)	0.33 (0.02)	0.62 (0.03)
	2k	10	0.91 (0.00)	0.39 (0.03)	0.43 (0.03)	0.76 (0.03)
		50	0.95 (0.00)	0.46 (0.02)	0.43 (0.02)	0.76 (0.02)
		100	0.95 (0.00)	0.39 (0.02)	0.43 (0.02)	0.76 (0.02)
T3*	7k	10	0.95 (0.02)	0.08 (0.04)	0.06 (0.03)	0.24 (0.05)
		50	0.97 (0.01)	0.08 (0.04)	0.05 (0.03)	0.22 (0.07)
		100	0.97 (0.02)	0.08 (0.04)	0.05 (0.03)	0.22 (0.06)
	4k	10	0.97 (0.01)	0.13 (0.03)	0.10 (0.01)	0.41 (0.03)
		50	0.97 (0.01)	0.12 (0.02)	0.10 (0.02)	0.40 (0.03)
		100	0.98 (0.01)	0.12 (0.02)	0.10 (0.02)	0.40 (0.03)
	2k	10	0.94 (0.00)	0.09 (0.01)	0.10 (0.01)	0.46 (0.02)
		50	0.98 (0.00)	0.15 (0.02)	0.11 (0.01)	0.47 (0.02)
		100	0.98 (0.00)	0.15 (0.02)	0.10 (0.01)	0.47 (0.02)

*The data under T3 were generated based on a 3D structure constructed using a GM cell (GSM3271347).
