# Supplementary material for: HiCImpute: A Bayesian hierarchical model for identifying structural zeros and enhancing single cell Hi-C data
Source: PLoS Comput Biol. 2022 Jun 13;18(6):e1010129. doi: 10.1371/journal.pcbi.1010129 (PMC9232133; doi:10.1371/journal.pcbi.1010129)
Supplement: S4 Table — We considered 2–6 clusters for HiCImpute-improved data and 2–4 clusters for the rest since the results did not indicate any need for a greater number of clusters. (PDF) [file pcbi.1010129.s016.pdf]

Table S4: K-means clustering results of L4 and L5 cells based on t-SNE embeded data. We considered 2-6 clusters for HiCImpute-improved data and 2-4 clusters for the rest since the results did not indicate any need for a greater number of clusters.

Observed (a). ARI=-0.003.

cell type	1	2
L4	76	55
L5	105	75

Observed (b). ARI=0.027.

cell type	1	2	3
L4	54	22	55
L5	41	64	75

Observed (c). ARI=0.031.

cell type	1	2	3	4
L4	42	12	22	55
L5	22	51	32	75

2DMF (a). ARI=-0.003.

cell type	1	2
L4	77	54
L5	105	75

2DMF (b). ARI=-0.003.

cell type	1	2	3
L4	38	54	39
L5	47	74	59

2DMF (c). ARI=0.003.

cell type	1	2	3	4
L4	24	30	54	23
L5	25	32	74	49

2DGK (a). ARI=-0.003.

cell type	1	2
L4	77	54
L5	104	76

2DGK (b). ARI=-0.003.

cell type	1	2	3
L4	54	36	41
L5	76	47	57

2DGK (c). ARI=-0.004.

cell type	1	2	3	4
L4	36	41	31	23
L5	47	57	46	30

RW3S (a). ARI=-0.003.

cell type	1	2
L4	76	55
L5	105	75

RW3S (b). ARI=-0.004.

cell type	1	2	3
L4	37	39	55
L5	51	54	75

RW3S (c). ARI=-0.004.

cell type	1	2	3	4
L4	29	26	39	37
L5	37	38	54	51

HiCImpute (a). ARI=-0.002.

cell type	1	2
L4	76	55
L5	106	74

HiCImpute (b). ARI=-0.002.

cell type	1	2	3
L4	55	0	76
L5	74	106	0

HiCImpute (d). ARI=0.506.

cell type	1	2	3	4
L4	0	55	76	0
L5	74	0	0	106

HiCImpute (d). ARI=0.392.

cell type	1	2	3	4	5
L4	0	55	0	0	76
L5	53	0	53	74	0

HiCImpute (e). ARI=0.336.

cell type	1	2	3	4	5	6
L4	55	32	0	44	0	0
L5	0	0	74	0	53	53
