# Supplementary material for: HiCImpute: A Bayesian hierarchical model for identifying structural zeros and enhancing single cell Hi-C data
Source: PLoS Comput Biol. 2022 Jun 13;18(6):e1010129. doi: 10.1371/journal.pcbi.1010129 (PMC9232133; doi:10.1371/journal.pcbi.1010129)
Supplement: S5 Table — (PDF) [file pcbi.1010129.s017.pdf]

Table S5: Computation time comparison of packages on three real datasets.

	HiCImpute	2DMF	2DGK	RW3S
GSE117874	6min	0.8s	1.5s	0.1s
GSE80006	2.5h	19s	15s	4s
scm3C-seq	17.3h	5min	4min	2min
