# Supplementary material for: HiCImpute: A Bayesian hierarchical model for identifying structural zeros and enhancing single cell Hi-C data
Source: PLoS Comput Biol. 2022 Jun 13;18(6):e1010129. doi: 10.1371/journal.pcbi.1010129 (PMC9232133; doi:10.1371/journal.pcbi.1010129)
Supplement: S6 Table — (PDF) [file pcbi.1010129.s018.pdf]

Table S6: Sparsity levels (percentages) of observed data and expected data.

Type	Sequence depth	Expected %zeros	Observed %zeros
T1	2K	4.48	37.37
	4K	4.48	14.08
	7K	4.48	10.26
T2	2K	4.48	40.32
	4K	4.48	19.29
	7K	4.48	8.53
T3	2K	4.48	39.56
	4K	4.48	19.55
	7K	4.48	9.12
