# Supplementary material for: HiCImpute: A Bayesian hierarchical model for identifying structural zeros and enhancing single cell Hi-C data
Source: PLoS Comput Biol. 2022 Jun 13;18(6):e1010129. doi: 10.1371/journal.pcbi.1010129 (PMC9232133; doi:10.1371/journal.pcbi.1010129)
Supplement: Attachment — Submitted filename: Response to Reviewers.pdf [file pcbi.1010129.s019.pdf]

Response to Reviewer 1's Comments

We thank the reviewer for their constructive comments. We have revised our manuscript accounting for their suggestions. Our point-to-point response is provided below each of the points (reproduced in italic). The revised texts in the highlighted version are in red, and page numbers refer to the revised version.

Xie et al. develop a Bayesian hierarchy model, HiCImpute, to improve single cell Hi-C (scHi-C) data and identify structural and sampling zeros, which focus on addressing an important problem in scHi-C analysis. The article is in general well-written and easy to read. My main concern lies in the insufficient description of HiCImpute. My suggestion is therefore to supplement related content to ensure a clearer description of this model.

Response: We thank you for your encouragement on this work, and we have revised the manuscript according to your comments, including additional description of HiCImpute. We provide point-to-point response below each of your specific suggestion.

Major suggestions:
1) What is Bayesian Hierarchical Model and why it could be applied to improve scHi-C data and identify structural zeros and sampling zeros. Above question should be described in the introduction.

Response: Following your suggestion, in the Introduction section, we have now provided a description of our Bayesian Hierarchical Model and stated why it is appropriate to use it for improving scHi-C data, especially for identifying structural zeros and imputing sampling zeros. In addition to making this clear in the introduction, we have also revised the Discussion section to provide some further insights (pages 6, 16, and 17).

2) Whether HiCImpute can enhance scHi-C data based on the identification of structural zeros and sampling zeros, that is, whether HiCImpute can only enhance the structural zeros but not sampling zeros.

Response. The idea is to balance the two competing goals, being able to identify structural zeros while also not labeling everything with an observed zero as being structural zeros. Both of these goals are factored into the Bayesian modeling in that both are parameters of interest in the posterior distribution and inference. Our ROC curves shows that we can indeed achieve both. We have now provided an explanation along this line in the Results section (pages 7, 10, and 11).

3) The bulk data used in HiCImpute is combining from single cells? Whether it can apply real bulk Hi-C data and is it necessary to use it. Besides, whether HiCImpute would introduce noise using bulk data as reference to improve Hi-C data. In other words, whether HiCImpute would enhance the signal which exist in bulk data but not in some single cell? In this situation, the heterogeneity of 3D structure may be neglected or incorrectly estimated.

Response. These are important points. If real bulk data are available and supplied by the user, then they are used directly in HiCImpute. Our K562 real data analysis used the real K562 bulk data since such are available. Our simulation study used "simulated bulk data" by combining many single cells (many more than the simulated scHiC data for analysis) to reflect "heterogeneity". The idea of incorporating bulk data, if such are available, is that, although heterogeneity of 3D structures is likely to be present in bulk data, there are also likely commonalities in a localized scale (neighborhoods) with much more accurate

observed counts (due to the order of magnitude higher sequencing depth), and their inclusion can help to delineate structural zeros and dropouts. In the hierarchical model, the single cells themselves play a very important, intertwining, role: if there are excess of counts in the neighborhood of a position in the 2D matrix among the single cells, then the information contained in the bulk data will be magnified; otherwise, it will be diminished. Nevertheless, we would like to note that bulk data are not required for HiCIMpute. Internally, if bulk data are not supplied, then single cells of the same major type (e.g. L4 in our prefrontal cortex example) will be pooled to form the "bulk data." These points are now more clearly presented in the Discussion and the Methods section (pages 16, 17, 19, and 20).

4) Whether HiCImpute could be applied to sample with large numbers of cells? What about the accuracy of improvement and time consumption?

Response. We have applied HiCImpute to a larger dataset with 311 cells of the prefrontal cortex (third application). As we elaborated in the Results and the Discussion sections, there was great agreement between the imputed and observed non-zeros, pointing to the accuracy of improvement (for the inference on the observed zeros), especially over the comparison methods (Supplementary Figures S5 and S6). We also discussed the time needed (Supplementary Table S5) to carry out the analysis for this much larger dataset and provided our rationale on why such a longer computational time is acceptable, especially giving the biological insights gained in the downstream analysis. We have further elaborated and highlighted these discussions in the revision (pages 13, and 17).

5) Whether the better performance of HiCImpute is related to the simulation procedure carried out in the text?

Response. Our simulation procedure is completely objective, which is not the same as our analysis procedure. We have also obtained much better, biologically appropriate, results for three real data studies. We have clarified this in the manuscript (page 8).

Minor suggestions:
1) P5: Taking on the challenging problems of separating the zeros into structural zeros and sampling zeros. What's the challenging problems specifically refers to? And why could Bayesian Hierarchical Model can address these problems?

Response. we have rephrased the sentence and provided the rationale for the appropriateness of the Bayesian hierarchical model for addressing these problems (pages 5 and 6).

2) It's better to adjust Figure 1 to show the schematic of the HiCImpute algorithm more clearly.

Response. We have modified Figure 1 according to your suggestion, and we have updated the text accordingly (page 7).

3) How to validate the cluster results are more accurate after data improved in Table2, especially in GM and K562 cell line, which are difficult to explain the biological meaning of cluster results? Number of clusters is not a sufficient criterion for judging the accuracy of classification.

Response. In table 2, since the underlying major cell types are known, our goal was to see whether cells belonging to the same known cell type are clustered together based on the original (before any improvement) or the improved data (according to several different improvement methods). We realize

that the table may be difficult to read; therefore, we have reformatted the table for clarity. Greater accuracy after data improvement is assessed as more cells of the same type are clustered together, and hope such information is more clearly seen in the reformatted Table 2. For example, for the first dataset containing cells of two types, GM and PBMC, with the original data (before improvement with any method), 13 GM and a significant portion (7) of the 18 PBMC cells were clustered together (C1). On the other hand, after data improvement with HiCImpute, all the GM cells were clustered into one group (C1); whereas of the 18 PBMC cells, 15 were clustered into another group (C2), although there were still 3 (which is much smaller than 7) that got grouped together with the GM cells (C1). This shows that the cluster results are more accurate with HiCImpute-improved data (Newly formatted Table 2).

4) There are a few format problems here and there. Besides, the abbreviations used in the text should be checked carefully, some words (e.g. structural zeros) are abbreviated repeatedly and the full name of these words appear after abbreviated.

Response. Thank you for pointing this out. We have checked through the entire manuscript trying to identify and correct any formatting problems that we can see. For the use of acronyms, we tried to be consistent. In occasions, we still use the full names for clarity.

P7L17: ...consider.It.. There should be a space between '.' and It.

Response. It's fixed.

P16L17: Among those observed 0's, some are true 0s (i.e. structural zeros, SZs) ..., irregular description.

Response. It's fixed.

5) It's better to unify the use of proper nouns (e.g. sampling zeros and dropouts) to avoid confusing readers.

Response. Thank you for the suggestion. We have now consistently use dropouts unless ambiguity occurs.

6) Please confirm the format requirement of the reference. The style of reference is not consistent with the article published recently.

Response. We checked recently published articles and modified the reference format to confirm to the journal style.

Response to Reviewer 2's Comments

We thank the reviewer for their constructive comments. We have revised our manuscript taking into account of their suggestions. Our point-to-point response is provided below each of the points (reproduced in italic). The revised texts in the highlighted version are in red, and page numbers refer to the revised version.

Xie et al. propose a method named HiCImpute to impute zeros in single-cell HiC data, while trying to differentiate between structural zeros and sampling zeros. The authors claim that HiCImpute outperforms three existing methods based on a set of simulated and real data studies. I agree this is an important research topic, but additional analyses or clarifications are in need to improve the validity of the method and strength of the claim.

Response. We thank you for your encouragement in noting that the topic being addressed in this paper is important. We have carried out additional analyses to further substantiate our findings.

The sequencing depth of the best K562 scHi-C data is 300,000. Why did the authors only consider sequencing depths of 2K, 4K, and 7K in the simulation? Can the authors show results on data with larger sequencing depths?

Response. We are very sorry for the confusion due to the lack of clarity in our description. The sequencing depth of 300,000 is for a genome-wide 2D matrix, while we only consider a submatrix of 61x61 in our simulation following an earlier study in the literature, and thus we rescaled the sequencing depth accordingly. We have clarified this in the revision (page 22).

The simulated contact matrix is 61 by 61. How is this dimension selected? This is quite small compared with the actual contact matrices.

Response. We followed the scheme devised in Han et al. (cited in the paper), which considered 61 loci on chromosome 1 for computational consideration, since a very large number of simulations was carried out. We have now clarified this in the revision (page 22).

What's the difference between data of T1, T2, and T3? The Method section mentions they are mimicking three K562 cells, but there are essentially the same cell type. It would be more interesting to evaluate the methods using simulated data of different cell types, to test the methods' performance given different data structures.

Response. We only borrowed the 3D structures constructed from the three K562 scHi-C data; however, the profiles of structural zeros and sampling zeros were different to simulate "subtype differences." Nevertheless, your point is well-taken, and we have simulated additional data based on the 3D structure from a GM cell (GSM3271347). Specifically, keeping T1 and T2 the same, we replaced the T3 data with simulated data based on the GM cell. Our results, provided as Supplementary Table S3, lead to the same conclusion as those based on the results presented in Table 1 in the main text: HiCImpute performs well and better than the other methods across the different simulated subtypes. We have modified the manuscript to include these results (page 11).

From the simulated data, the sparsity levels of the observed data and expected data are very similar (Figure 2). Based on the authors' description of single cell HiC data in Introduction, the observed data is supposed to be much sparser than expected data.

Response. This is indeed the case; the observed data are much sparser than expected data, especially for observed data with lower sequencing depth while the % zeros remain constant for the expected. It may be difficult to see this from Figure 2 as the heatmaps were used to only show patters. Therefore, we have now included the following table in the supplementary material and discuss this briefly in the main text (page 24 and Supplementary Table S6).

type	depth	% zeros of expected	% zeros of observed
I	2K	4.48%	37.37%
	4K	4.48%	14.08%
	7K	4.48%	10.26%
II	2K	4.48%	40.32%
	4K	4.48%	19.29%
	7K	4.48%	8.53%
III	2K	4.48%	39.56%
	4K	4.48%	19.55%
	7K	4.48%	9.12%

"For the three comparison methods, an observed zero is identified as SZ if its imputed value is less than 0.5." Is this threshold applied to count data or normalized data? If normalized data is used, how is the normalization done?

Response. For the comparison methods, each single cell was analyzed separately because these methods do not borrow information from other single cells. Therefore, the threshold of 0.5 was applied to the count, not normalized, data. We have now clarified this in the revision (page 9).

Can the authors clarify if HiCImpute is the only method that requires *bulk data among the four compared methods? This is not very clear in the current manuscript.*

Response. Indeed, HiCImpute is the only method among the four compared that can make use of information from bulk data, if such is available, although not *required*. We have now clarified this in the manuscript (page 4).

It seems that the proposed Bayesian hierarchy model requires prior information of cell types or clusters, since the model assumes that the K cells have the same pattern of SZs. Therefore, it is not clear how HiCImpute is used to improve clustering analysis in the real data applications. Was HiCImpute applied to single cell data without using any cell type information? How does the model distinguish different cell types during the imputation process?

Response. The goal of this work is on improving data quality assuming we know the major cell types, with the hope that, with improved data, we may be able to detect subtypes. As demonstrated in our three data applications, the cell type information in each case was known. We further demonstrated

that, with the third example, we were indeed able to find subtypes within each of the two known types, consistent with the literature using other data modalities. We have provided discussion on this in the revision (page 17).

For the mean of the Gamma distribution, how is the neighborhood defined? Is this done in a data-adaptive manner? Does this range affect the imputation performance?
Can the authors explain why this mean is defined based on the ratio between bulk and single-cell data?

Response. In our initial submission, we consider a single neighborhood within a fixed width (two bins) from each position in the 2D matrix, following 2DMF. Taking your comment into consideration, we carried out a sensitivity analysis to evaluate the effect of the selected neighborhood. Specifically, we considered two other neighborhoods, either with 8-closest neighbors (one bin away in all directions) or a neighborhood with first- and second-degree neighbors (spatial statistics terminology). The results, presented in Supplementary Table S2, show that the outcomes are rather insensitive to the choice of a neighborhood. In addition to the addition of Table S2, we have also added discussion on this in the revised manuscript (page 11).
For the mean of the Gamma distribution, after the neighborhood is selected, we opted to set it using both the bulk and the single cell data. Specifically, we first use the normalized neighborhood-mean of the bulk data to set the initial value as such data are likely to be more reliable given its much larger sequencing depth. This value is then modified by the single cell data as such data also carry information on the overall mean, and the modification is done in such a way that if there are excess of counts in the neighborhood of a position in the 2D matrix among the single cells, then the information contained in the bulk data will be magnified; otherwise, it will be diminished. This point is now more clearly presented in the Methods section (pages 19 and 20).

Does the posterior distribution have a closed-form representation? If yes, this should be added to the main text. It would also be helpful to list the full model (in math notations) after explaining individual model assumptions in Method.

Response. Unfortunately, the posterior distribution does not have a closed form because the normalizing constant cannot be found analytically. We have now explained this more clearly in the revision (page 20) and we have provided the entire posterior distribution in the supplementary materials due to the length of the math formula (over 3/4 of a page).
